# Microheterogeneity in Liquid Water Associated with Hydrogen-Bond Cooperativity-IR Spectroscopic and MD Simulation Study of Temperature Effect

**DOI:** 10.3390/ijms26115187

**Published:** 2025-05-28

**Authors:** Paulina Filipczak, Marcin Kozanecki, Joanna Szala-Rearick, Dorota Swiatla-Wojcik

**Affiliations:** 1 Department of Molecular Physics, Lodz University of Technology, Zeromskiego 116, 90-924 Lodz, Poland; paulina.filipczak@p.lodz.pl (P.F.); marcin.kozanecki@p.lodz.pl (M.K.); 2 Institute of Applied Radiation Chemistry, Lodz University of Technology, Zeromskiego 116, 90-924 Lodz, Poland; j.szala.rearick@gmail.com

**Keywords:** IR spectroscopy, MD simulation, liquid water, hydrogen-bonding interaction, hydrogen-bond network, structure, hydrogen-bond connectivity

## Abstract

Structural microheterogeneity arising from the cooperative nature of hydrogen bonding is a critical yet often overlooked factor in the mechanistic understanding of physicochemical and biological processes occurring in aqueous environments. MD simulations using a potential that accounts for molecular flexibility and directional interactions revealed inhomogeneity arising from patches of continuously connected, four-bonded molecules embedded within a less ordered, space-filling hydrogen-bond network. The size of these patches follows a statistical distribution that is strongly temperature-dependent. With increasing temperature, the average size of the patches decreases, whereas the contribution of molecules forming the inter-patch zones becomes more pronounced. The nature of microheterogeneity is evidenced by temperature-dependent changes in the asymmetry of calculated power spectra as well as in the measured IR absorption within the stretching, bending, and combination band regions. A novel method for band analysis incorporates the calculation of skewness and a mirroring procedure for more accurate determination of FWHM of asymmetric bands. Discontinuities in the temperature dependence of spectral parameters observed within the 5–80 °C range correspond to the thermodynamic anomalies of liquid water. We show that structural microheterogeneity persists near 100 °C, suggesting that aqueous processes are better described by statistical distributions than by uniform models. Molecular simulations and IR spectroscopy offer key insights into these distributions.

## 1. Introduction

**Figure 1:**
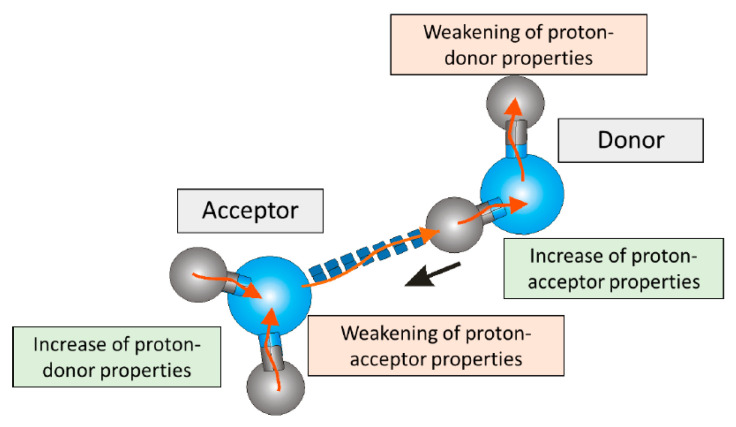
Scheme illustrating the influence of hydrogen bond formation on the proton-donor and proton-acceptor character of water molecules. The H-donor molecule has increased electron density in its ‘lone pair’ region, encouraging the formation of additional H bonds with H-donor molecules, while the protons on the H-acceptor have decreased electron density, making them more susceptible to binding with H-acceptors.

Water is the most common and simultaneously the most mysterious solvent on Earth. With its numerous anomalous and unique properties—such as volume contraction upon melting, a minimum in isothermal compressibility under normal conditions, high surface tension, a density maximum at 4 °C, and decreased viscosity under pressure—water is far from being a simple liquid [1,2,3]. It is now beyond any doubt that water’s unique behavior results from hydrogen bonds (H-bonds) and the ability of a single H_2_O molecule to form two H-donor and two H-acceptor bonds. The formation of a single H-bond between two water molecules induces changes in their electron clouds, which increase the ability for further hydrogen bonding [4], as illustrated in Figure 1. This behavior partially explains the willingness of water molecules to form larger supramolecular structures, as networks of H-bonds show cooperativity, resulting in stronger interactions than expected from simple pairwise additivity.

In natural Ih ice, the four H-bonded neighbors of each water molecule are positioned in a tetrahedral arrangement, forming a hexagonal lattice [1]. The cooperativity of H-bonds is not restricted only to ice but is also observed in the liquid state. Experimental studies on water and ice using X-ray spectroscopy and X-ray Raman scattering, reviewed by Nilsson and Pettersson [3], identified two local structural motifs: low-density liquid (LDL), characterized by tetrahedral H-bonds, and high-density liquid (HDL), which interconvert with temperature changes. The LDL-like structure is stabilized by the cooperativity of tetrahedrally-bonded molecules and is thus enthalpically favored at lower temperatures. In contrast, HDL is structurally less ordered and forms a more constrained environment, allowing for librational vibrations, and is thus entropically favored at elevated temperatures. Based on scattering experiments and MD simulations, Stanley and coworkers [5,6,7,8] and Huang et al. [9] developed a picture describing water in the liquid state as a temperature-dependent fluctuating equilibrium between two types of local structures: tetrahedral-like patches of the density lower than the global one and denser, less ordered H-bonded molecular networks.

The connectivity of H_2_O molecules via H-bonds studied by MD simulation using the flexible potential showed that the microheterogeneity associated with patches, in which the individual water molecule participates twice as a proton donor and twice as a proton acceptor, decreases upon heating but may persist in the pressurized liquid at temperatures above 100 °C [10]. More recent calculations of time autocorrelation functions of H-bonded molecular pairs indicated slower breaking and faster reforming of temporarily broken H-bonds in the presence of large patches [11]. The diminishment of the patch-like inhomogeneity upon increasing temperature was evidenced by a sharp decrease in the continuous and intermittent lifetimes of H-bonds. In this study, MD simulation using the same model potential, accounting for molecular flexibility and directional interactions of hydrogen atoms, was employed to compute power spectra of inter- and intra-molecular vibrations, i.e., respectively hindered translations and hindered rotations (librations), and stretching and bending vibrations. The results of the computational part of our study are presented in Section 2.2, together with an overview of the calculated structural properties and an analysis of the molecular organization into hydrogen-bonded nets and patches.

The objective of the experimental part, presented in Section 2.1, was to determine whether infrared (IR) spectroscopy can be used as a probe of structural inhomogeneity in liquid water and to evaluate the influence of temperature on this inhomogeneity. The measurements were carried out for liquid water under standard pressure and at temperatures ranging from 5 to 80 °C. Previously, vibrational spectroscopy has been widely applied to the study of the gas and liquid phases of water, as well as crystalline and amorphous ices [12,13]. These measurements mostly focused on the OH stretching modes. The OH stretching frequency is very sensitive to the intermolecular distances, and thus, reflects different local arrangements of molecules. Here, we have investigated changes in the spectral parameters of the bending, stretching, and combination bands, representing short-range (local) and high-range (global) interactions. Using a novel method for spectra analysis that incorporates the calculation of skewness and a mirroring procedure for more accurate determination of the FWHM of asymmetric bands, we identified discontinuities in the temperature dependence of spectral parameters, discussed in Section 3. These discontinuities correspond closely with known thermodynamic anomalies of liquid water.

The existence of local structures challenges the view that liquid water is a homogeneous reaction medium. Since microheterogeneity inevitably affects the behavior of solutes and solute impact on water–water interactions, a characterization of the temperature dependence of the spatial and temporal scale of structural inhomogeneity is essential for understanding chemical processes in aqueous environments. As postulated by Swiatla-Wojcik [14], the production of molecular hydrogen in the bimolecular reaction of two hydrated electrons (e^−^_aq_) at ambient and elevated temperatures is inherently connected with the presence of patches. This example shows that a mechanistic view, which takes into account structural inhomogeneity, is important to resolve the effect of temperature on the kinetics of processes involved in green energy applications. The results of this study suggest that aqueous processes up to 100 °C are more effectively characterized by statistical distributions than by uniform models. Moreover, the integration of molecular simulations with IR spectroscopy offers valuable insights into these distributions.

## 2. Results

### 2.1. IR Spectroscopy

**Figure 2:**
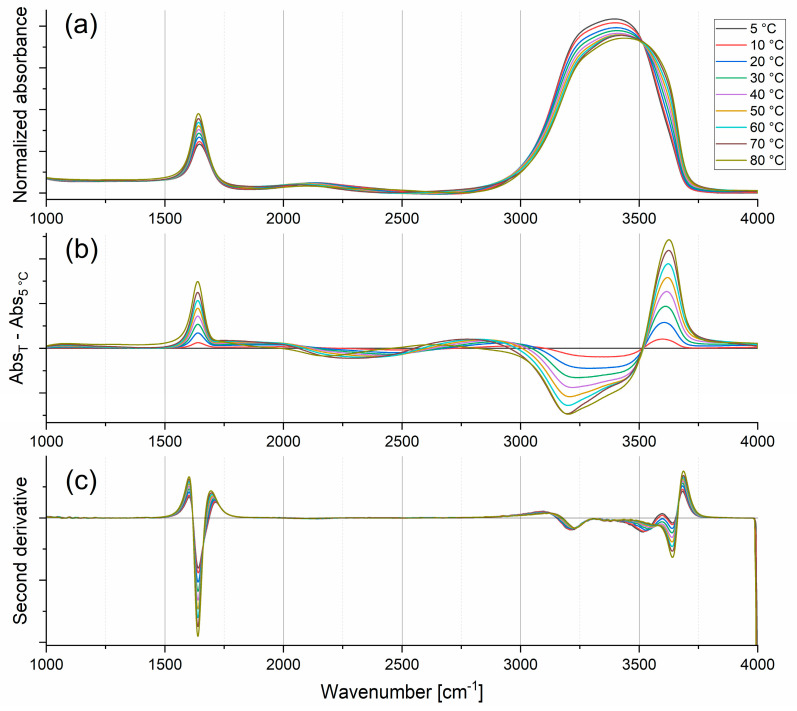
Normalized IR absorption spectra at different temperatures (**a**). The difference spectra in relation to the spectrum obtained at 5 °C (**b**). The second derivatives of normalized spectra (before and after second derivative calculations spectra were smoothed) (**c**).

An indirect way of probing the intermolecular network is to look at the intramolecular modes of water molecules since their oscillator forces are sensitive to the interactions of the molecules with their surroundings. It is observed that in a set of connected molecules, a change in the oscillator force of an individual bond induces correlated changes in the oscillator forces of the neighboring bonds (whether intramolecular or intermolecular). In the case of liquid water, infrared spectroscopy is an adequate tool to precisely detect such variations in bond oscillations, due to the large transition dipole moment of water [15]. Figure 2a presents the IR absorption spectra of liquid water in the mid-IR range, where three of the main vibrational bands are present. All spectra were normalized to the total integral intensity (surface area under the spectrum). The difference spectra were obtained by subtracting the water spectrum at the lowest measured temperature (5 °C) from the spectrum obtained at the specified temperature (see Figure 2b). To precisely determine the band maxima, the second derivatives of the spectra were calculated (see Figure 2c).

The dominating feature of the IR absorption spectrum of water is a broad band located at 3400 cm^−1^, with shoulders at about 3250 cm^−1^ and a weak one at around 3600 cm^−1^, assigned mainly to OH stretching vibrations (with a small contribution from the bending mode overtone). A strong and narrow band at 1645 cm^−1^ corresponds to a bending mode. There is a small but significant combination band of the bending and libration modes at about 2125 cm^−1^, also known as the ‘association band’ [16]. Fundamental libration modes (restricted rotations–rocking motions, imposed by H-bonding), not visible in Figure 2, occur below 1000 cm^−1^. A major libration–L2—has its maximum at 680 cm^−1^, whereas a minor one–L1—at 395 cm^−1^ [17].

In liquid water, the molecular stretch vibrations shift to higher frequencies with increasing temperature (as H-bonding weakens, the covalent O-H bonds strengthen, causing them to vibrate at higher frequencies), while the molecular bending vibration peak becomes both narrower and stronger [18]. In an H-bonded water system not perturbed by any factors (such as high temperature or the presence of solutes, especially ionic), strong interactions between water molecules and strong cooperativity of stretching vibrations are present. This collectivity of the OH-stretching vibrational modes blocks bending vibrations. With the rise in temperature and a decrease in the number of H-bonds in the system, bending vibrations of water molecules become more active, as there are more degrees of freedom for this motion. The differences between stretching and bending vibrations are due to the increased importance of intermolecular H-bonding at lower temperatures, which tends to reduce intermolecular bending while encouraging stretching. Thus, in the extreme non-H-bonded state, the ‘dangling’ O-H bond stretch frequency at surfaces is 3697 cm^−1^ [19]. A rise in the temperature also lowers the intensity of the main stretching bands, while the absorbance of the component around 3600 cm^−1^ is increasing. The combination band’s absorbance value and wavenumber both reduce with increasing temperature.

#### 2.1.1. Stretching Modes

**Figure 3:**
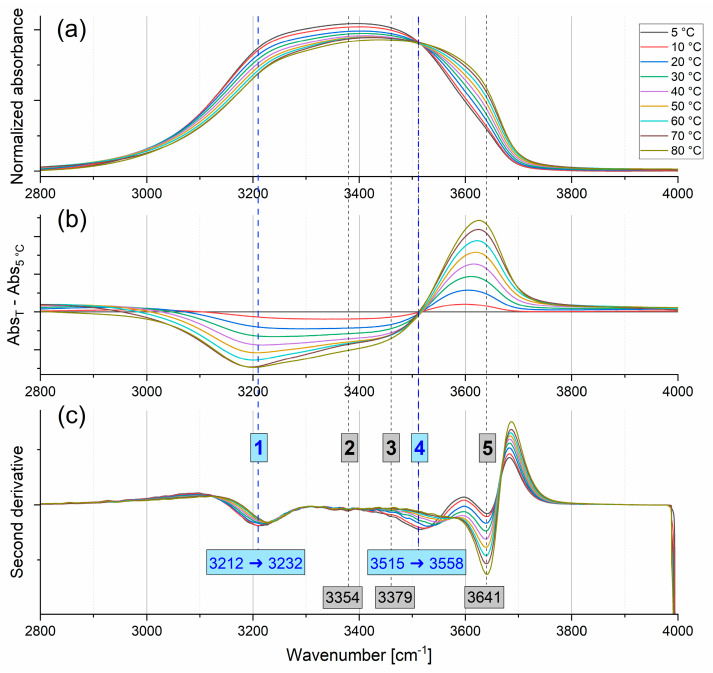
Normalized IR absorption spectra in the OH stretching vibration region at different temperatures (**a**). The difference spectra in relation to the spectrum obtained at 5 °C (**b**). The second derivatives of normalized spectra (**c**).

The first step of the detailed analysis was the investigation of the broad band in the OH stretching vibration region (3000–3800 cm^−1^). This wide band is almost featureless, which makes it challenging to perform a precise analysis of its non-distinguished components. However, in addition to the standard procedure in presenting the temperature evolution of the spectra (difference spectra), the second derivative method was also used (see Figure 3). The second derivative method is useful for determining the number of overlapping components within complex bands and has already been used for the analysis of the water IR absorption spectrum [20,21,22]. In the difference spectra, one can clearly distinguish negative and positive bands divided by a clear isosbestic point at 3520 cm^−1^. An isosbestic point distinguishes at least two fractions of structures present in the system: more and less ordered. The population of more ordered structures decreases with increasing temperature, while that of less ordered structures increases. In the negative broad band below the isosbestic point (which decreases with increasing temperature), two components can be clearly distinguished: the main one at about 3200 cm^−1^ and the other one at around 3400 cm^−1^. In the part above the isosbestic point, a single band at around 3620 cm^−1^ increases with an increase in temperature. A quick look at the second derivative spectra (Figure 3) shows that the analysis of results by difference spectra may be misleading, as it does not reveal all components of the broad OH stretching vibrations band.

**Figure 4:**
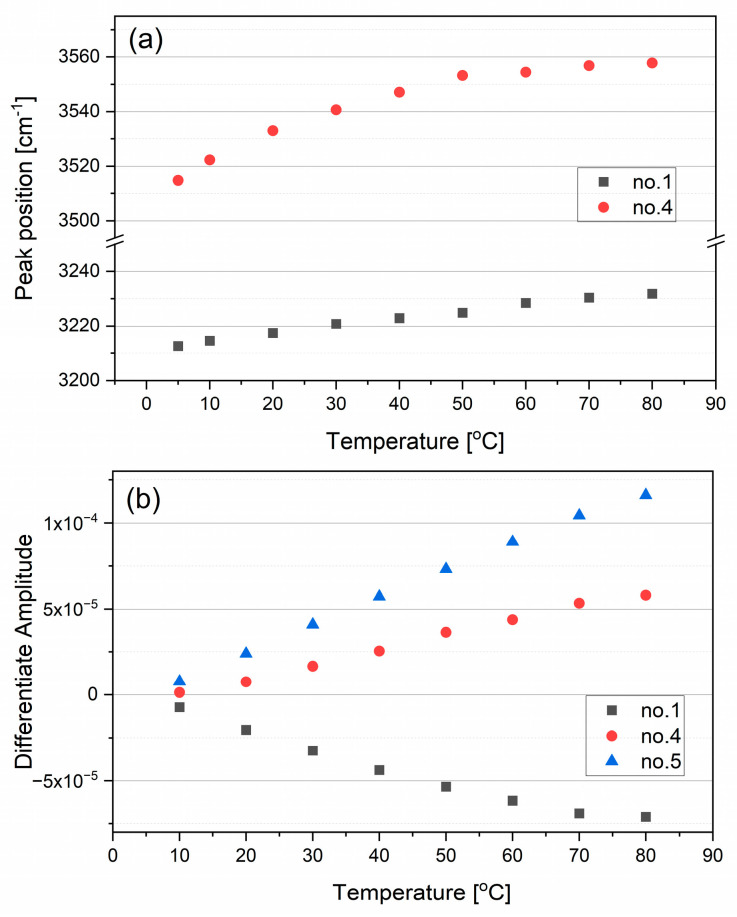
Changes in the peak positions of the main components in the second derivative spectra in the OH stretching vibration region with increasing temperature (**a**), and changes in the differentiate amplitude values of the main components (**b**).

Dashed lines (numbered from 1 to 5) in Figure 3c indicate the distinguished components from the second derivative spectrum at 5 °C. Blue numbers corresponds to the bands whose position changes with increasing temperature. The numbers for bands 1, 4, and 5 correspond to obvious positions of second derivative local minima. Band no. 1 nicely aligns with the minimum of a component in the difference spectrum. The position of band no. 4 at 5 °C agrees with the position of an isosbestic point, then shifts to higher wavenumbers with increasing temperature. Comparing the position of this band in the difference spectra, one can notice that with the rise in temperature, it is added to the positive component above the isosbestic point. Numbers 2 and 3 are assigned to bands whose precise positions cannot be obtained. Band no. 3 appears as a shoulder of band no. 4. Additionally, it aligns with the position of one of the components in the difference spectrum. Band no. 2 is very weak across all temperature spectra. The noisy signal in this spectral region also makes it difficult to estimate its position accurately. Changes in the positions of the bands with increasing temperature, as observed in the second derivative spectra for bands no. 1 and 4, are presented in Figure 4. The position of the band at 3640 cm^−1^ remains constant (the deviation from the average value is not higher than 1 cm^−1^, which is within the margin of error). The positions of bands no. 1 and 4 shift to higher wavenumbers as temperature increases—a significantly larger shift is observed for band no. 4, from 3515 to 3558 cm^−1^, compared to band no. 1, which shifts from 3212 to 3232 cm^−1^.

#### 2.1.2. Bending Mode

**Figure 5:**
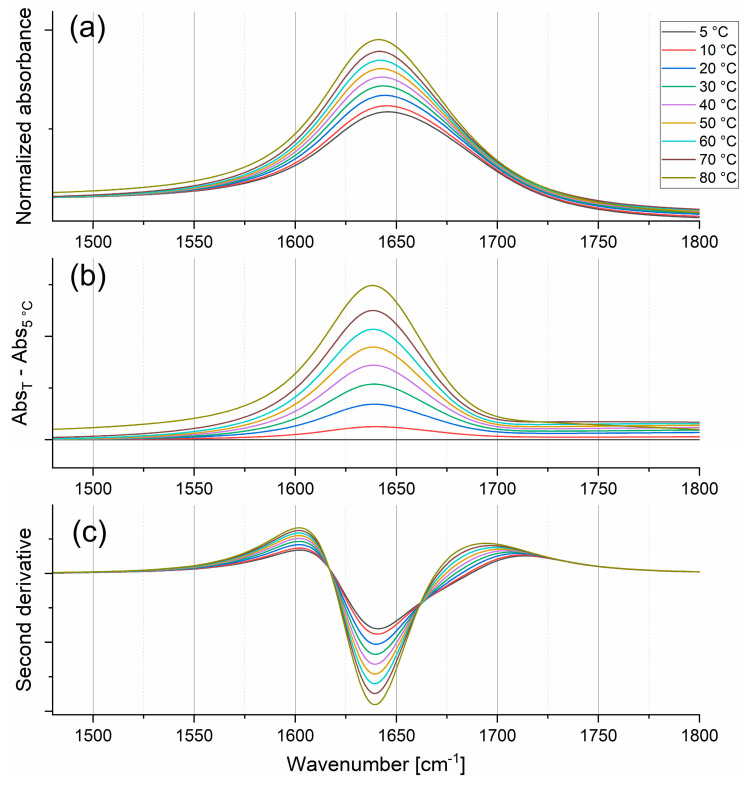
Normalized IR absorption spectra in the HOH bending vibration region at different temperatures (**a**). The difference spectra in relation to the spectrum obtained at 5 °C (**b**). The second derivatives of normalized spectra (**c**).

An increase in temperature causes changes also in the H_2_O bending vibrational mode (see Figure 5), corresponding to the vibrations of the H-O-H angle in liquid water. The maximum of the bending, δ_H-O-H_, band is observed around 1645 cm^−1^ at room temperature, and its position shifts to lower wavenumbers with increasing temperature. Walrafen et al. [23], based on the analysis of polarized Raman spectra, proposed to deconvolute this band into two Gaussian components [23]—one broader with a lower amplitude, representing less organized water structure, while the second, narrower and more intense, relates to a better-organized water structure. The molecular interpretation of both components was made considering the opposite trends in intensity changes of both lines observed with increasing temperature. The broader peak became more intense and narrower, while the relative intensity of the narrower band decreased with increasing temperature. Analysis of the FTIR spectra in the bending region is problematic, mainly due to the high asymmetry of the δ_H-O-H_ band and the different baseline levels at the left and right sides of the maximum. These two facts strongly limit the application of classical statistical treatment methods such as deconvolution. Moreover, the second derivatives of the obtained spectra did not confirm the presence of two individual lines within the δ_H-O-H_ band (see Figure 5c). Therefore, a novel approach to describe the changes in the bending band of water was proposed herein (see description and Figures S1–S3 in Supplementary Materials Section). Briefly, the band was shifted along the “Wavenumber axis” to place the band maximum at 0. Next, the band was divided into two parts (negative and positive ones), and these parts were reflected about the “Intensity axis”. Finally, two symmetrical bands were obtained, which could be statistically analyzed separately. Such an approach allowed us to analyze the left and right branches of the water banding band independently. As the total FWHM of the δ_H-O-H_ band, the sum of half the FWHM of both artificially created symmetrical lines was assumed.

**Figure 6:**
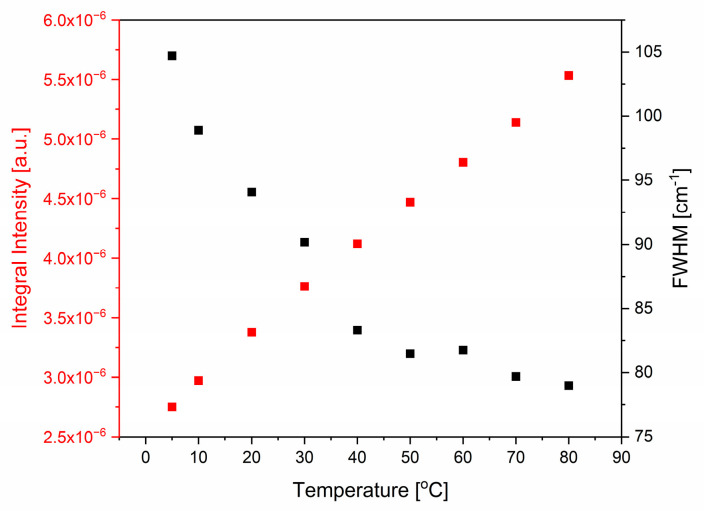
The relations between total full width at half maximum and integral intensities of H_2_O bending mode and temperature.

The temperature dependence of the total FWHM and the integral intensity of the δ_H-O-H_ band are shown in Figure 6. The bending motions are favored at high temperatures due to a decrease in the cooperativity between water molecules (a smaller number of H-bonds blocking molecules’ bending vibrations). Thus, the integral intensity of the bending band increases linearly with temperature. The opposite tendency is observed for the total FWHM. This effect is also consistent with the general picture of water structure. The decay (or reduction) of the average water patch size (see Section 2.2.2) with temperature leads to a limitation of long-range interactions and a better localization of bending modes. As a result, the vibrational density of states for the bending mode becomes narrower.

#### 2.1.3. Combination Modes

**Figure 7:**
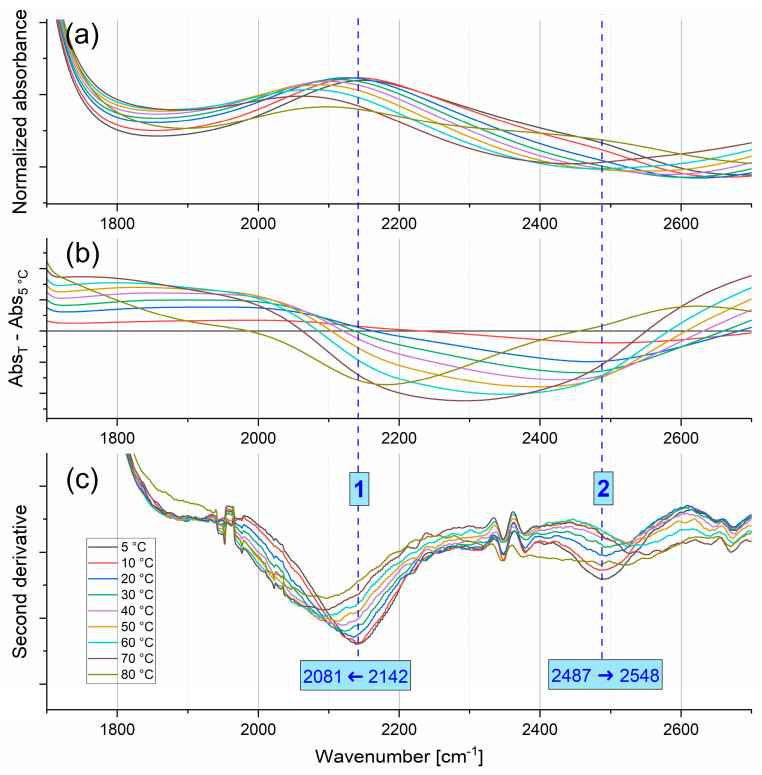
Normalized IR absorption spectra in the combination band range at different temperatures (**a**). The difference spectra in relation to the spectrum obtained at 5 °C (**b**). The second derivatives of normalized spectra (**c**). Dashed lines represent positions of the bands at 5 °C. The annotation of the no. 2 peak final shift is presented at 70 °C.

**Figure 8:**
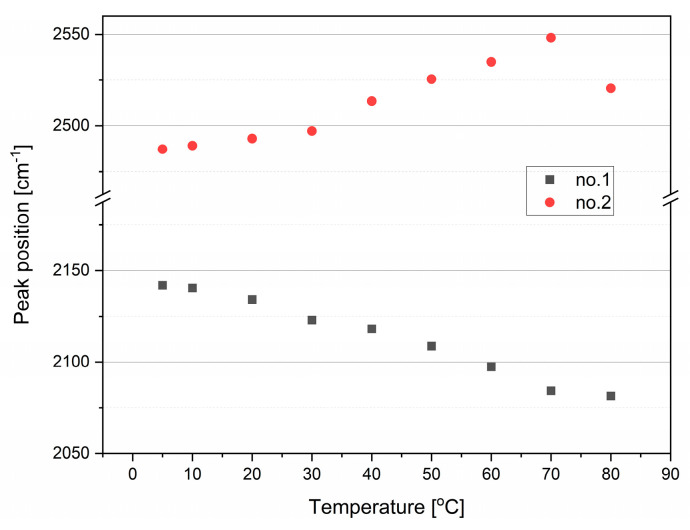
Changes in the peak positions of the main components in second derivative spectra in the combination band range with increasing temperature.

Between 1800 and 2600 cm^−1^, combination modes are visible in the FTIR spectrum of water—see Figure 7. The second derivative method allowed us to distinguish two main absorption lines sensitive to temperature. Interestingly, the positions of the maxima of the observed lines shift in opposite directions (see Figure 8). The low-frequency mode shifts from 2142 cm^−1^ at 5 °C to 2081 cm^−1^ at 80 °C, while the high-frequency component shifts from 2487 at 5 °C to 2548 cm^−1^ at 70 °C. Giuffrida et al. [16] wrote that the origin of the combination band (also called association band) is “still a puzzle”. The source of the combination band may be a combination of libration and bending modes or the overtones of librations. The major libration in water, L2, has its maximum at c.a. 680 cm^−1^, whereas a minor one, L1, appears near to 395 cm^−1^ [17]. A simple combination of δ_H-O-H_ and L2 modes should result in the band position at c.a. 2325 cm^−1^; a combination of overtones of L2 and L1 modes should give a line at 2150 cm^−1^, while a combination of δ_H-O-H_ and the L1 overtone would appear at 2435 cm^−1^. Independently of the identification of the combination band origin, the strong contribution of the L2 mode to the low component of the combination band is evident, as the positions of the L1 and δ_H-O-H_ modes are only weakly dependent on temperature, while the L2 mode is very sensitive to temperature changes [17]. More ambiguous and surprising is the shift of the high-frequency component of the combination band to higher wavenumbers. Verma et al. showed [24] that the combination band may be a measure of the collective vibrations of water molecules, and that agents destructive to the water structure (temperature or chaotropic salts) shift this band to lower wavenumbers, while structure-forming agents such as kosmotropic salts cause the opposite shift. However, it is necessary to underline that Verma et al., as well as other authors [24,25,26], did not distinguish particular components of the combination band but treated it as a whole.

The temperature characteristics of the main component positions of the combination band in water exhibit two “kinks”. The first one is between 30 and 40 °C, where the dependencies become stronger, while the second one is at 70 °C. In general, the distance between the components increases as the temperature increases (an exception is the spectrum recorded at 80 °C). These changes are described in more detail in the Section 3.

### 2.2. MD Simulation

Classical molecular dynamics (MD) simulation is a deterministic method for analyzing the trajectories of particles (atoms, molecules) in phase space. The behavior of a system of interacting particles is determined by numerically solving Newton’s equations of motion, where the forces between particles and their potential energies are calculated using interatomic potentials or molecular mechanical force fields. According to the ergodic hypothesis, the macroscopically observed properties of a system described by the microcanonical ensemble are equivalent to the time average over a long interval. Therefore, time-ordered data obtained from NVE simulations can be used to calculate spatial and velocity time correlation functions, which can be related to the structural, dynamical, and thermodynamical properties of the simulated system. Details on the model potential and the simulation procedure used in this study are provided in Section 4.2.

#### 2.2.1. Radial Distribution Functions

**Figure 9:**
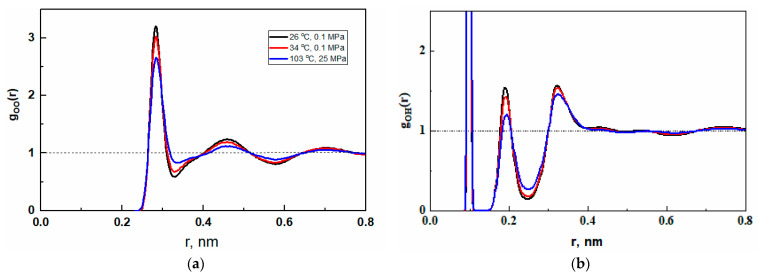
Partial radial distribution functions for water under standard pressure at 26 °C (black), and 34 °C (red), and under 25 MPa at 103 °C (blue). (**a**) The oxygen–oxygen RDFs. (**b**) The oxygen–hydrogen RDFs.

Radial distribution functions describe the spatial correlation of molecules. According to the adopted three-center flexible model of the water molecule, we can describe the spatial correlation of oxygen atoms, as well as the intramolecular and intermolecular correlations between the oxygen atom and the hydrogen atom. The oxygen–oxygen (g_OO_) and oxygen–hydrogen (g_OH_) RDFs are presented in Figure 9. As can be seen, the positions of maxima and minima do not change, but the fluctuations become less pronounced with increasing temperature. This indicates a reduction in the range of spatial correlation characteristic of the tetrahedral arrangement of molecules.

The intermolecular g_OH_ precedes g_OH_ and shows that the nearest molecule distance is shorter than the sum of the Van der Waals radii of the oxygen and hydrogen atoms (0.27 nm). These features, confirming hydrogen bonding interactions, are observed at all studied temperatures.

#### 2.2.2. Hydrogen Bond Network

The structural properties of the hydrogen bond network have been analyzed in terms of short-range and long-range networking. The H-bond definition used includes the following criteria [27]: (i) the pair interaction energy, E < −8 kJ/mol; (ii) the distance between the hydrogen atom of the H-donor and the oxygen atom of the H-acceptor, R_OH_ < 0.25 nm; (iii) the inclination of the OH bond of the H-donor to the line connecting the oxygen atoms, α < 30 °C.

**Figure 10:**
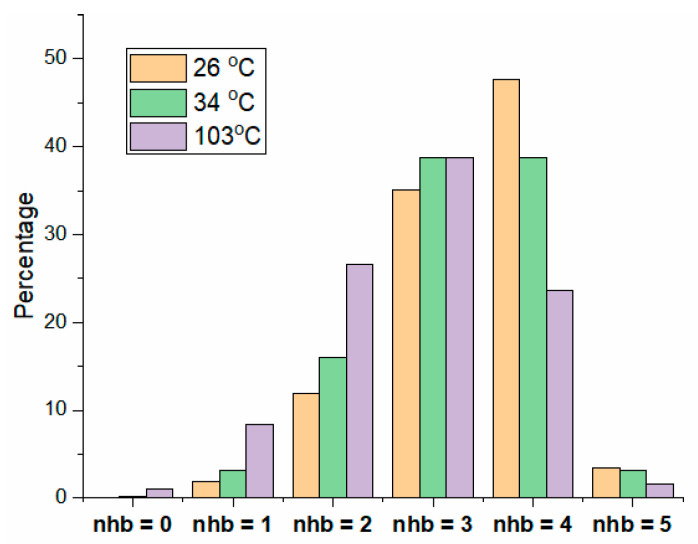
Percentages of molecules forming various numbers of hydrogen bonds (n_hb_): water under standard pressure at 26 and 34 °C, and under 25 MPa at 103 °C. The contribution of bifurcated H-bonds (n_hb_ = 5) is also shown. Bifurcated H-bonds include cases where one H-donating molecule is bound to two H-bond acceptors, or where H-bond donors are bound to a single H-bond acceptor. The mean numbers of H-bonds per molecule <n_hb_> are 3.4, 3.2, and 2.8, at 26, 34, and 103 °C, respectively.

The histograms in Figure 10 illustrate the short-range networking within the H-bond network. They depict the statistical distribution of the number of H-bonds formed by individual molecules at 26, 34, and 103 °C.

**Figure 11:**
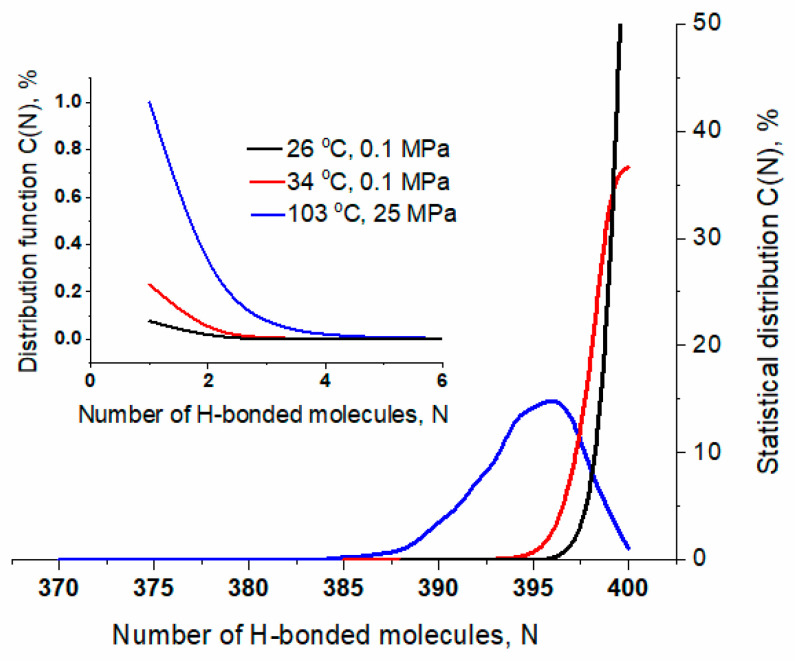
The normalized statistical distribution of the number of molecules N linked within the H-bonded network, calculated at 26 °C (black), 34 °C (red), and 103 °C (blue). The mean number <N> is higher than 400 at both 26 and 34 °C, and is equal to 390 at 103 °C.

The dominant presence of three and four-bonded molecules is observed at both 26 and 34 °C. At 103 °C, the contribution of four-bonded molecules is clearly smaller, and molecules forming three H-bonds predominate. The histogram also shows the absence of unbound molecules (n_hb_ = 0) and, on the other hand, a noticeable contribution of bifurcated H-bonds, which significantly influence the stability of the molecular structure. The statistical mean <n_hb_> slightly decreases with increasing temperature within 26–34 °C, and more significantly at 103 °C. However, <n_hb_> = 2.8 is still above the percolation threshold [7,8,10], where initially disconnected clusters of H-bonded molecules become connected. The presence of a large, space-filling network is confirmed by statistical distributions of the number of molecules connected by a path of H-bonds (see Figure 11).

**Figure 12:**
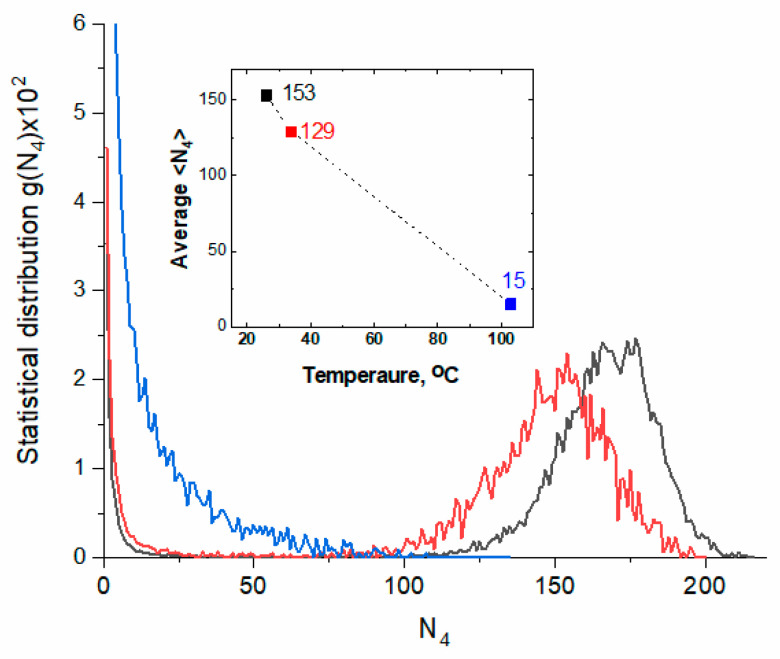
The normalized distribution of the patch size, defined as the number of connected four-bonded molecules, calculated for 26 °C (black), 34 °C (red), and 103 °C (blue). Inset: average size <N_4_> as a function of temperature.

The inhomogeneity of the continuous H-bond network is evidenced by the statistical distribution of patch sizes, defined as the number of four-bonded molecules (N_4_) linked together within a single supramolecular structure (see Figure 12).

At both 26 and 34 °C, the distribution function g(N_4_) is bimodal, with the right peak indicating the presence of large patches. At 103 °C, g(N_4_) decreases monotonically, and the calculated mean value indicates a significant reduction in patch size.

#### 2.2.3. Power Spectra

**Figure 13:**
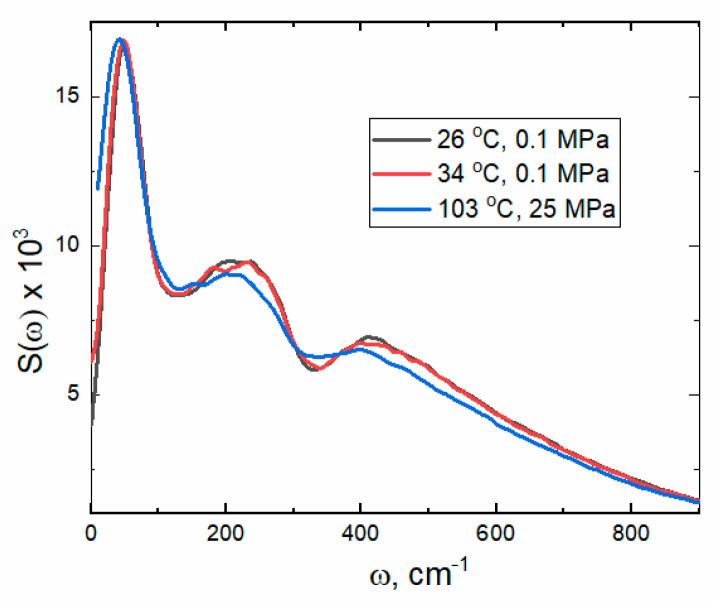
Calculated spectral density S(ω) (power spectrum) of hindered translations and hindered rotations (librations).

**Figure 14:**
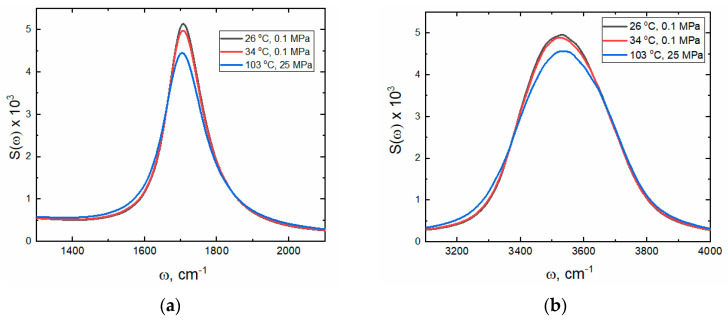
Calculated vibrational power spectra of water at 26 °C (black), 34 °C (red), and 103 °C (blue). (**a**) Spectral density profiles of the H-O-H bending. (**b**) Spectral density profiles of the O-H stretching. Uncertainty in the band position is 10 cm^−1^.

The use of the flexible model potential makes it possible to monitor changes in the molecular geometry caused by intermolecular interactions [28]. The linear response theory relates the absorption cross section to the Fourier transform of the ensemble average autocorrelation function of the dipole moment $<C_{\mu \mu }\left(t\right)>$ of the vibrating molecule [1], as follows:

(1) $$S(\omega )=\frac{1}{2\pi }\int_{-\infty }^{\infty }<C_{\mu \mu }(t)>e^{-i\omega t}dt$$

where ω is the angular frequency. For real $<C_{\mu \mu }\left(t\right)>$ the spectral density (the power spectrum) S(ω) can be calculated as the sum of the velocity correlation functions of the hydrogen (H) and oxygen (O) sites of the H_2_O molecule $<C_{V_{H}V_{H}}>$ and ${<C}_{V_{O}V_{O}}>$ [29]. An anharmonic coupling between intra- and intermolecular modes due to the H-bonding interaction can be observed as shifts in the power spectra of hindered translations, librations (Figure 13), and vibrations (Figure 14).

A band around 50 cm^−1^ was identified in Raman spectroscopic experiments [30] and interpreted as O^…^O^…^O intermolecular bending vibration or a restricted translation of water molecules in a rigid H-bond network stiffened by the presence of large patches. Hindered rotations or intermolecular stretching in a rigid network manifest as two bands near 200 and 400 cm^−1^. A slight decrease in the intensities of these bands at 103 °C is consistent with the reduction in patch size.

The power spectra of the intramolecular vibrations depicted in Figure 14 are clearly asymmetric. Skewness coefficients calculated for the bending bands within 1400–2000 cm^−1^ are 1.38 (26 °C), 1.35 (34 °C), and 1.23 (103 °C). The positive values indicate a right-skewed asymmetry, i.e., a longer ‘right tail’.

The stretching bands are also right-skewed, but their asymmetry is much smaller. The skewness coefficients within 3150–3950 cm^−1^ are 0.40 (26 °C), 0.39 (34 °C), and 0.32 (103 °C). It is important to note that the calculated vibrational bands are noticeably less asymmetrical at 103 °C.

The asymmetry of the spectral bands suggests that the water molecules are experiencing local environments with varying strengths and configurations of H-bonds, as reflected by the distribution of patches (see Figure 12). The decomposition of the power spectra into two Gaussian bands confirms stronger and weaker H-bonding (see Table 1 and Figure S4).

## 3. Discussion

The experimental and simulation results consistently indicate structural microheterogeneity associated with the cooperativity of H-bonds. The structural characteristics of water are highly dependent on the temporal and spatial scales at which they are examined. The static FTIR spectroscopy used in this study, along with the simulation results, provides insight into the diffusion-averaged structure.

The effect of temperature on the dynamic character of microheterogeneity is evidenced by changes in the asymmetry of the calculated power spectra and in the measured IR absorption within the stretching, bending, and combination band regions. The very small volume of the water molecule and the small distance between neighboring water molecules in the liquid phase result in strong vibrational coupling. Therefore, changes in particular vibrations of the water molecule could not be analyzed separately. Yu et al. [31] reported different ways of vibrational coupling in liquid water, including stretching, bending, and libration modes. Time-resolved IR measurements revealed that intermolecular stretch-to-stretch coupling is much stronger than intermolecular bend-to-bend (intramode) coupling. It is also interesting to mention that the vibrational energy of the bending mode is mainly released to the libration modes of the same molecule (intramolecular energy transfer), resulting in strong bend–libration coupling. This means that the librational modes play an important role in the relaxation of the excited bending mode. Contrarily, the stretching modes of water are coupled to the vibrations of neighboring water molecules [31].

**Figure 15:**
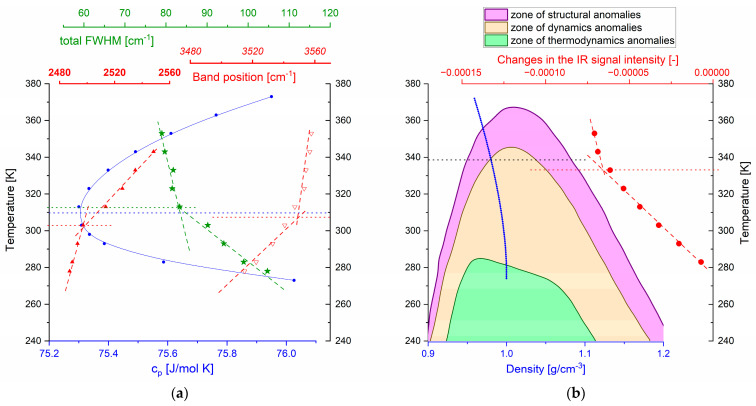
The temperature dependences of the total FWHM of bending mode (green stars) and the band components positions of the OH stretching mode (no. 4) (red empty triangles) and the combination mode (no. 2 in Figure 7c) (red full triangles), shown against the background of the thermal dependency of the isobaric specific heat, c_p_ (blue dots) [37] (**a**). Changes in the intensity (signal amplitude) of the OH stretching mode (component no. 1), shown against the background of water anomalies (zones of occurrence of particular types of anomalies are shown in different colors: pink—zone of structural anomalies, yellow—zone of dynamics anomalies, green—zone of thermodynamics anomalies) [36], and the experimentally determined thermal evolution of water density [38] (**b**).

The observed changes in the character of spectral parameters correspond well with the anomalies of water properties (see Figure 15). The minimum in the temperature dependence of the isobaric specific heat, c_p_, located at 35 °C (308 K) [32,33,34,35], coincides with changes in the slopes of the temperature dependencies observed for (i) the peak position of the stretching mode labeled as peak 4 (see also Figure 3c and Figure 4); (ii) the peak position of the combination mode labeled as 2 (see also Figure 7c and Figure 8); (iii) the total FWHM of the bending mode (see Figure 6). These parameters are related to different vibrational modes, proving their strong coupling. Both c_p_ (representing changes in enthalpy) and absorption spectroscopy in the middle range of IR reflect intermolecular interactions. Above the minimum of c_p_, the stretching mode becomes less sensitive to temperature, while the combination band shows the opposite trend. This can be explained by considering the temperature impact on the patch-like microheterogeneity of the water structure (see Figure 12). With increasing temperature, the patches become smaller and better separated, and, as a consequence, the cooperativity of water molecule dynamics (including vibrations) decreases. The OH stretching vibrations, which mainly provide information about the local dynamics of the water molecule, become less sensitive to the temperature increase. On the other hand, the combination band, connected to more global dynamics of water and reflecting potential coupling of librations and bending modes, is more sensitive to temperature changes.

The decrease in the population of water molecules involved in patches becomes steeper above 65 °C (Figure 15b). As the FTIR experiments were performed under standard pressure, the water density changes with temperature according to the blue curve. Close to 65 °C, this curve crosses the boundary of the dynamics anomalies zone [36]. The coincidence of this point with the kink in the temperature dependence of changes in the amplitude of the stretching mode labeled in Figure 4 as no. 1 is clearly seen.

**Figure 16:**
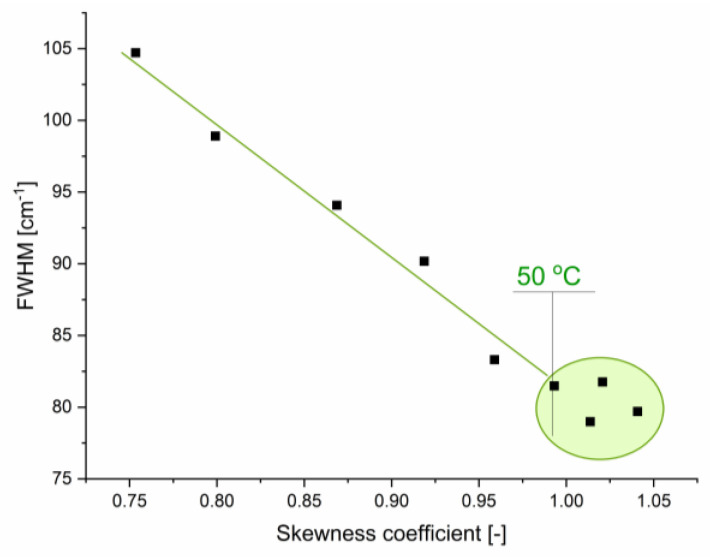
The relation between the total FWHM and the skewness coefficient of δ_H-O-H_ band.

It is also interesting to analyze the bending mode in more detail, as it is highly asymmetric, and its asymmetry changes with temperature. One of the parameters useful for determining band asymmetry is the skewness coefficient. Figure 16 shows the total FWHM as a function of the skewness coefficient for the bending band. Up to ca. 45 °C, a linear correlation between these two parameters is visible, suggesting a common origin of changes in these parameters. At higher temperatures, the correlation disappears, demonstrating the influence of different factors. In this context, it is also noteworthy that at 46 °C under standard pressure, a minimum in isothermal compressibility is observed [35,39,40].

**Figure 17:**
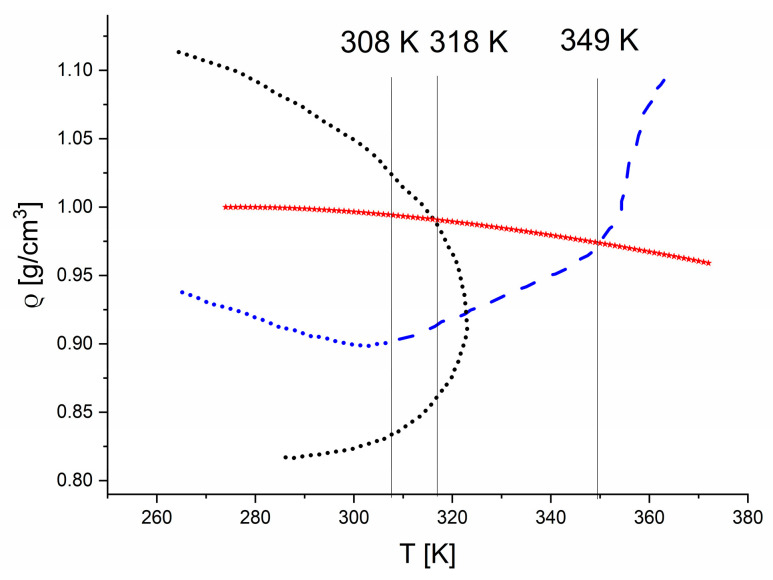
Positions of the kinks (thin vertical lines) in the studied thermal dependencies of FTIR line parameters shown in Figure 15, plotted on the background of selected temperature dependences related to κ_T_ and density in the ρ–T plane. The Λ_max_(T) and Λ_min_(T) curves (blue dots and blue dashed line, respectively) correspond to the maxima and minima of thermal compressibility, while the Δ_max_ (black dots) line represents the density maxima on the water isochores. The Λ_min_(T), Λ_max_(T), and Δ_max_(T) curves were determined based on MD calculations for the ST2 water model [41]. Experimentally determined thermal evolution of water density is also shown (red points) [38].

Characteristic kinks observed in the temperature dependences of spectral parameters of the bending, combination, and stretching bands (see Figure 15a) also correlate with theoretical predictions reported by Poole et al. [41], as shown in Figure 17 on the ρ–T plane (where ρ is water density). The red line represents the conditions of the FTIR experiments performed here. At 349 K, the density line crosses the Λ_min_(T) curve, along which the minimum of thermal compressibility (κ_T_) occurs, i.e., (∂κ_T_/∂T)_P_ = 0. Close to this specific temperature, a kink in the temperature dependence of the changes in the amplitude of stretching mode no. 1 was found. Moreover, the disappearance of the correlation between the total FWHM and the skewness coefficient observed at 318 K corresponds to the crosspoint of the Δ_max_ line (representing the density maxima on the water isochores at 318 K) and the density curve. Finally, the kinks in the temperature dependences of the FTIR band parameters in Figure 15a were found at 308 K. Below this temperature, minima in thermal compressibility no longer occur; instead, the κ_T_ maxima appear. Our FTIR results are also in agreement with the results presented by other authors [41,42,43].

The picture of liquid water built of highly ordered patches distributed within a less ordered environment is consistent with the model of amorphous solid states (glasses), which assumes the presence of highly cohesive domains dispersed in a less cohesive medium [44]. Assuming that glasses can be treated as frozen liquids that (at least partially) preserve the molecular organization of liquids, the patch-based model seems to be a very adequate representation of water structure in the liquid state. The patches correspond to highly cohesive domains characterized by superior molecular ordering and a large number of water molecules forming four or bifurcated hydrogen bonds with neighboring molecules, whereas the less cohesive medium is associated with molecules constituting the inter-patch zones. At low temperatures, the distribution of patch size is bimodal, indicating a high population of large patches. As the temperature rises, the patches shrink while the inter-patch zones expand, leading to a shortening of the lifetime of hydrogen bonds [11] and alternations in water dynamics, as revealed by the spectroscopic measurements.

## 4. Materials and Methods

### 4.1. Experimental Method

The water used in the experiments was of HPLC grade purity, purchased from Sigma Aldrich (Burlington, MA, USA). Absorption spectra in the mid-infrared (MIR) range were recorded using a Nicolet Fourier-Transform iS50 spectrometer (Thermo Scientific, Waltham, MA, USA). Temperature experiments were performed using the Falcon Mid-IR Transmission Accessory (PIKE Technologies, Madison, WI, USA), where absorption is measured in the transmission mode. Zinc selenide (ZnSe) windows, with a broad transmission spectral range (20,000–500 cm^−1^) and a sample absorption path length of 15 μm, were selected for the measurements. Information about the experimental equipment can be found at the following websites: https://studylib.net/doc/8290686/thermo-scientific-nicolet-is50-ft-ir-spectrometer, https://www.piketech.com/wp-content/uploads/PDS/transmission/PIKE-Technologies_Mid-IR-Falcon.pdf (accessed on 16 May 2025). Temperature experiments were performed in the range of 5–80 °C, with a set point accuracy of ±0.5%. Measurement parameters were as follows: resolution −2 cm^−1^; number of scans per measurement—256 scans. Each sample spectrum measured at a specified temperature was background-corrected using a spectrum obtained for an empty cuvette at the same temperature.

### 4.2. Computational Method

NVE ensemble MD simulations of water at 298 K (25 °C) and 310 K (37 °C) under standard pressure of 0.1 MPa, as well as for pressurized liquid at 373 K and 25 MPa (100 °C, 25 MPa), were performed using the three-site modified central force flexible potential with partial charges q_O_ = −0.66e and q_H_ = +0.33e, centered on the oxygen (O) and the hydrogen (H) sites, respectively [27]. The intermolecular part of this potential describes Coulombic and short-range non-Coulombic interactions [29] between the molecular sites, O-O, H-H, and H-O, whereas the intramolecular interactions are expressed via the power series of internal coordinates, as proposed by Carney et al. [45]. In all simulations, the cubic box contained 400 water molecules. The length of the cubic box was calculated based on the experimental densities: 0.997 g·cm^−3^, 0.994 g·cm^−3^, and 0.969 g·cm^−3^, respectively. The initial configuration was obtained by random placement of water molecules in the cubic box and assuming an equilibrium gas-phase geometry for each molecule. Initial velocities were sampled from the Boltzmann distribution. The equations of motion were integrated using the Verlet algorithm and assuming a simulation step of 0.1 fs. Long-range and short-range non-bonding interactions were treated by the Ewald summation and the shifted-force potential method, respectively. Equilibration was performed by simulated annealing and cooling. To observe no trend in temperature, the equilibration stage required ca. 10^6^ time steps. The length of the production run was 50 ps. The positions and velocities of the molecular sites were stored every 1 fs. The stability of the total energy was 10^−6^ < ΔE/E < 10^−5^. The statistical mean and the standard deviation of the temperature of the equilibrated systems were (299.0 ± 5.8) K, (306.9 ± 5.3) K, and (376.3 ± 6.8) K, respectively. These systems are denoted in the text as 26 °C, 34 °C, and 103 °C, respectively.

## 5. Conclusions

This study demonstrates that structural microheterogeneity—driven by hydrogen-bond cooperativity and manifested as highly ordered patches embedded within less ordered regions—persists in liquid water even at temperatures approaching 100 °C. With increasing temperature, the average size of the patches decreases and the contribution of molecules forming the inter-patch zones becomes more pronounced, leading to changes in the dynamics of hydrogen-bonded molecules. This behavior is evidenced by changes in the asymmetry of calculated power spectra and by variations in the measured absorption within the stretching, bending, and combination band regions. Using a novel method for spectra analysis that incorporates the calculation of skewness and a mirroring procedure for more accurate determination of the FWHM of asymmetric bands, we identified discontinuities in the temperature dependence of spectral parameters that align with known thermodynamic anomalies of liquid water.

This study suggests that processes occurring in aqueous environments are better described by statistical distributions than by uniform models. In this context, the role of water microheterogeneity in heterogeneous systems emerges as both a promising and challenging direction for future research.

## Figures and Tables

**Table 1:** Features of two bands resulting from Gaussian decomposition of the calculated spectral profiles S(ω) *.

	Bending			Stretching		
System	26 °C 0.1 MPa	34 °C 0.1 MPa	103 °C 25 MPa	26 °C 0.1 MPa	34 °C 0.1 MPa	103 °C 25 MPa
1ω_max_	1709	1706	1704	3551	3558	3638
1W_1/2_	92	82	88	333	303	240
1S_max_	0.0032	0.0024	0.0021	0.0045	0.0042	0.0023
2ω_max_	1724	1721	1715	3463	3443	3480
2W_1/2_	250	191	204	154	165	251
2S_max_	0.0016	0.0021	0.0019	0.0007	0.0009	0.0030

* Maximum position (1ω_max_, 2ω_max_), half-width (1W_1/2_, 2W_1/2_), and height at the maximum (1S_max_, 2S_max_) of the two Gaussian bands.

## Data Availability

Data are available from the corresponding author upon request.

## Supplementary Materials

The following supporting information can be downloaded at: https://www.mdpi.com/article/10.3390/ijms26115187/s1.
