# Supplementary material for: Microheterogeneity in Liquid Water Associated with Hydrogen-Bond Cooperativity-IR Spectroscopic and MD Simulation Study of Temperature Effect
Source: Int J Mol Sci. 2025 May 28;26(11):5187. doi: 10.3390/ijms26115187 (PMC12155183; doi:10.3390/ijms26115187)
Supplement: Supplementary file 1 [file ijms-26-05187-s001.zip › ijms-3635257-supplementary.pdf]

Supplementary Materials

Microheterogeneity in Liquid Water Associated with Hydrogen-Bond Cooperativity-IR Spectroscopic and MD Simulation Study of Temperature Effect

Paulina Filipczak [1], Marcin Kozanecki [1], Joanna Szala-Rearick [2] and Dorota Swiatla-Wojcik [2,*]

[1] Department of Molecular Physics, Lodz University of Technology, Zeromskiego 116, 90-924 Lodz, Poland; paulina.filipczak@p.lodz.pl (P.F.); marcin.kozanecki@p.lodz.pl (M.K.)
[2] Institute of Applied Radiation Chemistry, Lodz University of Technology, Zeromskiego 116, 90-924 Lodz, Poland; j.szala.rearick@gmail.com
* Correspondence: dorota.swiatla-wojcik@p.lodz.pl

Analysis of the full width at half maximum (FWHM) of the bending mode

Due to the asymmetric behaviour of the bending mode, and also the presence of the step (difference in the baseline level before and after the band) different approach for these data analysis had to be done. For the analysis of FWHM changes in the bending mode along with the temperature rise, the data were treated in the following way: i) normalised (to the surface area in the range 1000–4000 cm^{-1}); ii) data in the range 1480 – 1800 cm^{-1} where normalised in this manner that the wavenumber at which the maximum absorption occurs is treated as zero; iii) data on the left and right side of the maximum were mirrored separately to obtain two symmetrical bands. Example of this data treatment for the spectrum of the water sample at 5 °C is presented in Figure S1.

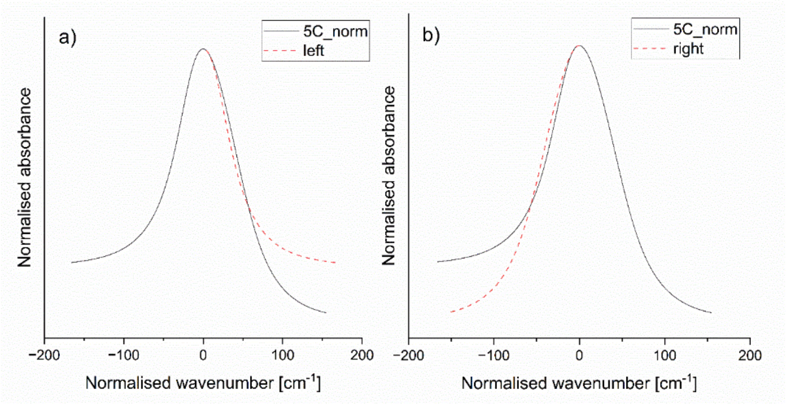

Figure S1. An example of the data treatment for the spectrum of the water sample at 5 °C.

The comparison of obtained symmetrical bands for two water spectra acquired at different temperatures (5 and 70 °C) is presented in Figure S2.

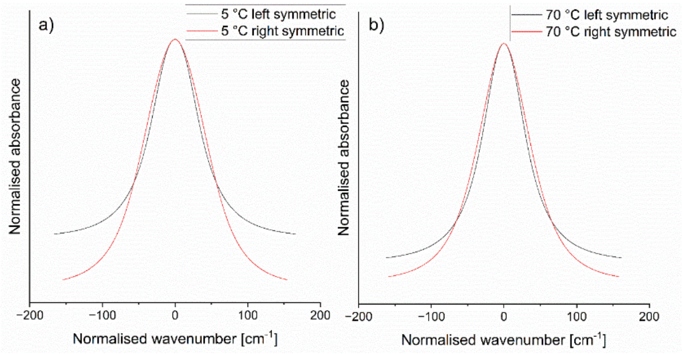

Figure S2. The obtained symmetrical bands from the mirroring procedure, examples for the water spectra acquired at 5 and 70 °C.

Peak fitting with the use of PsdVoigt2 (Pseudo Voigt) formula revealed the changes in the FWHM values of both symmetrical bands with the increase of the temperature. To estimate the FWHM changes in the original asymmetrical band following formula was applied:

$$FWHM_{combined} = \frac{1}{2}\, FWHM_{left} + \frac{1}{2}\, FWHM_{right} \qquad \text{(S1)}$$

where $FWHM_{left}$ and $FWHM_{right}$ are FWHM of the symmetrical bands created by the mirroring (see Figure S3).

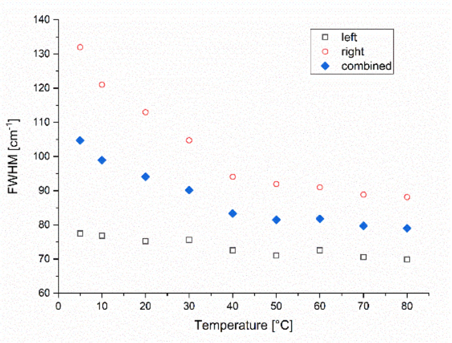

Figure S3. Comparison of the FWHM′ values for the obtained symmetrical bands (left and right) and combined FWHM calculated from eq. (S1).

Exemplary Decomposition of the Calculated Power Spectra

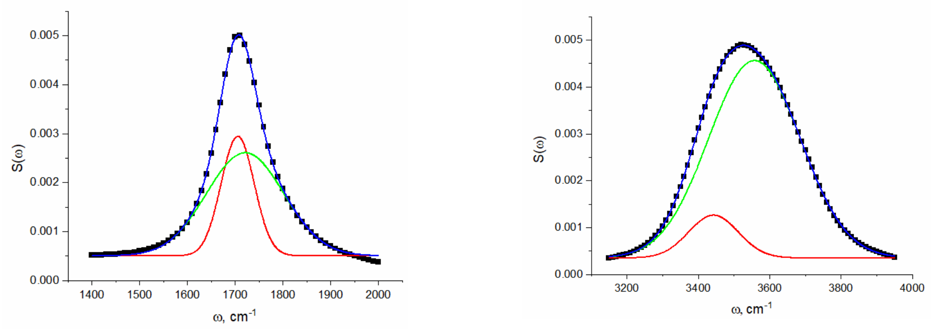

(**a**) (**b**)

Figure S4. Decomposition of the calculated vibrational power spectra of water at 34 °C (blue line + points) into **two Gaussian profiles** shown by red and green lines. (**a**) Bending. (**b**) Stretching.
